# Coexistent Relapsing Polychondritis and Clinically Amyopathic Dermatomyositis: A Rare Association of Autoimmune Disorders

**DOI:** 10.1155/2023/3719502

**Published:** 2023-04-11

**Authors:** Rafael A. Ríos-Rivera, Luis M. Vilá

**Affiliations:** Division of Rheumatology, University of Puerto Rico Medical Sciences Campus, San Juan, PR, USA

## Abstract

Relapsing polychondritis (RPC) is an uncommon autoimmune systemic disease characterized by recurrent inflammation of the cartilage tissue. It can occur alone or in association with other autoimmune diseases, vasculitis, or hematologic disorders. However, the association of RPC with dermatomyositis is extremely rare. Herein, we present a case of a 38-year-old man who developed concurrent RPC and clinically amyopathic dermatomyositis (CADM) manifested by auricular chondritis, nasal chondritis, polyarthritis, gottron papules, fingertip papules, skin biopsy consistent with dermatomyositis, and positive antimelanoma differentiation-associated gene 5 (MDA5) antibodies. RPC features resolved with corticosteroids, but CADM manifestations were resistant to corticosteroids, cyclophosphamide, azathioprine, and hydroxychloroquine. Subsequent therapy with rituximab was effective to control CADM manifestations. This case highlights the importance of recognizing CADM as part of the autoimmune diseases linked with RPC and maintaining a high level of awareness to initiate effective therapy to avoid the long-term complications associated with these conditions.

## 1. Introduction

Relapsing polychondritis (RPC) is an uncommon autoimmune systemic disease characterized by recurrent inflammation of the cartilage tissue mostly involving the ears, nose, respiratory tract, and joints as well as proteoglycan-rich tissues such as the heart valves, blood vessels, and eyes [1]. Epidemiologic studies from the United Kingdom, Hungary, and the United States (US) show an annual incidence estimated in 0.7–3.5 per million people, with the highest incidence reported in the US [2–4]. RPC afflicts both sexes equally and the usual age of onset is between 40 and 60 years [1, 2]. It can occur alone or in association with other diseases including autoimmune diseases, systemic vasculitis, or hematologic disorders in about 30%–35% of cases [2, 5–7]. The most common autoimmune diseases seen in RPC are rheumatoid arthritis and autoimmune thyroiditis. On the other hand, the association with inflammatory myopathy is rare.

Dermatomyositis (DM) is an autoimmune disease characterized by skin lesions and muscle inflammation with an annual incidence of approximately 1 per 100,000 people [8]. Clinically, amyopathic dermatomyositis (CADM) is a distinct form of DM in which cutaneous manifestations are present in the absence of muscle involvement. Recent studies have estimated the annual incidence of CADM in 0.2 per 100,000 people [8]. It is more common in females and any age can be affected. Like RPC, DM may be associated with other rheumatic diseases such as systemic sclerosis, systemic lupus erythematosus, mixed connective tissue disease, and less frequently rheumatoid arthritis and Sjögren's syndrome [9]. However, few reports have documented the coexistence of RPC with DM and, to the best of our knowledge, none with CADM. Herein, we present a young adult man who concurrently developed RPC and CADM.

## 2. Case Presentation

A 38-year-old-man with Hashimoto's thyroiditis presented to the emergency department with a 2-month history of progressive fatigue, unintentional weight loss of 20 pounds, unquantified fever, myalgias, polyarthralgia, swelling of hands and feet, pain and swelling of ears, pain and redness of nose, hoarseness, and erythematous rash over his knuckles, elbows, and knees. He had no Raynaud phenomenon, skin tightening, photosensitivity, oral ulcers, sicca symptoms, muscle weakness, cough, shortness of breath, dyspnea on exertion, palpitations, abdominal pain, diarrhea, paresthesia, dizziness, vertigo, seizures, or cognitive impairment. He had no family history of autoimmune diseases.

**Figure 1:**
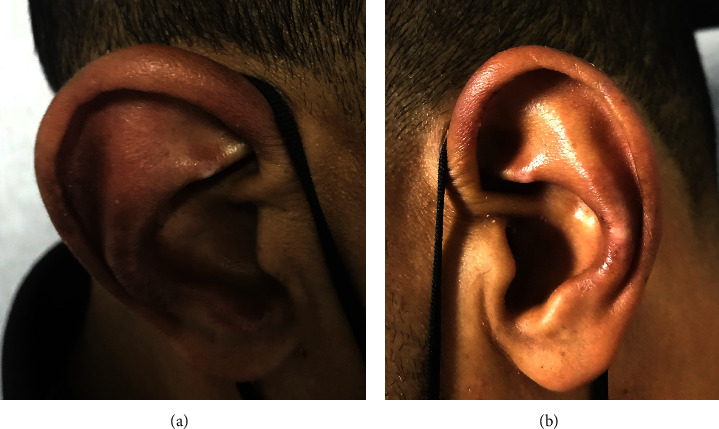
(a) Right and (b) left auricular involvement characterized by purplish redness and swelling of the pinna but sparing the lobules.

**Figure 2:**
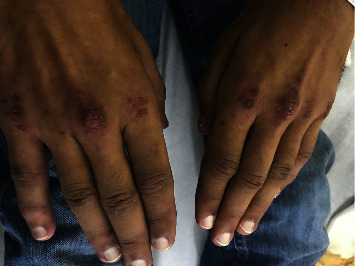
Erythematous, flat-topped, hyperkeratotic papules on the dorsal metacarpophalangeal joints bilaterally (gottron papules).

On physical examination, he had a temperature of 36.3°C, heart rate of 89 bpm, blood pressure of 114/70 mmHg, and respiratory rate of 18 rpm. He had swelling and tenderness of both auricles sparing the lobules (Figure 1) and erythematous and tender nose. Skin examination showed gottron papules (Figure 2), erythematous plaques overlying the knees and elbows, bilateral nontender subcutaneous nodules in the olecranon area, and patchy alopecia. Musculoskeletal exam revealed swelling and tenderness of the left second proximal interphalangeal joint and the second, third, and fourth metatarsophalangeal joints bilaterally. Muscle strength was normal in all extremities. Lymphadenopathy was not observed. Pulmonary, cardiovascular, abdominal, and neurologic examinations were unremarkable.

Laboratory tests disclosed a white blood cell count of 4,820 *μ*L, platelet count of 286,000 *μ*L, and hemoglobin of 12.2 g/dL. Serum creatinine was normal at 0.76 mg/dL. Aspartate aminotransferase and alanine aminotransferase were slightly elevated at 64 U/L (normal range, 0–40 UL) and 55 U/L (normal range, 0–41 UL), respectively. Alkaline phosphatase, total bilirubin, creatine phosphokinase, and aldolase levels were normal. Urine analysis revealed no proteinuria, hematuria, pyuria, or urinary casts. He had mild elevation of the Westergren sedimentation rate at 36 mm/hour and C-reactive protein at 11.4 mg/L (normal range <5 mg/L). Antinuclear antibodies (ANA) were elevated at 1 : 40 titer (the homogeneous pattern). Anti-dsDNA, anti-Smith, antiribonucleoprotein, anti-Ro, anti-La antibodies, anticardiolipin antibodies (IgA, IgG, and IgM), and anti-*β*2-glycoprotein I antibodies (IgA, IgG, and IgM) were not detected. Lupus anticoagulant was negative and serum C3, C4, and cryoglobulin levels were normal. The rheumatoid factor and anti-CCP antibodies were negative. Antineutrophil cytoplasmic antibodies, antimyeloperoxidase antibodies, and antiproteinase-3 antibodies were undetected. The autoimmune myositis panel disclosed positive antimelanoma differentiation-associated gene 5 (MDA5) antibodies. Antiaminoacyl tRNA synthetase (Jo-1, PL-7, PL-12, EJ, and OJ), antisignal recognition particle (SRP), anti-Mi-2 (alpha and beta), antitranscription intermediary factor 1*γ* (TIF-1*γ*), and antinuclear matrix protein 2 (NXP-2) antibodies were negative.

**Figure 3:**
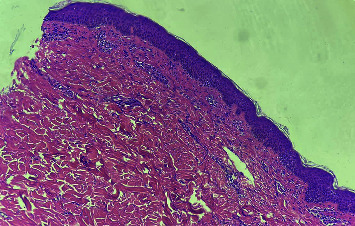
Biopsy of the posterior aspect of the right elbow showing perivascular lymphohistiocytic infiltration without lichenoid changes (hematoxylin and eosin stain, 100x).

Chest x-rays were normal. X-rays of the hands showed no osteopenia, joint space narrowing, or marginal erosions. Computed tomography of the chest, abdomen, and pelvis was unremarkable. No myopathic changes were observed on electromyography. Transthoracic echocardiography depicted the normal left ventricular function and no pericardial effusion or valve abnormalities. Skin biopsy of the elbow disclosed lymphocytic inflammatory infiltrates at the dermal-epidermal junction basement membrane and perivascular lymphocytic infiltrates without lichenoid changes with positive mucin in alcian blue stain consistent with DM (Figure 3).

Our patient was diagnosed with RPC and CADM. The diagnosis of RPC was made using the classification proposed by MacAdam and Damiani et al. [10, 11]. On the other hand, the diagnosis of CADM was made by the presence of rash typical of DM (e.g., gottron papules), biopsy-confirmed cutaneous manifestations of DM, and no clinical evidence of proximal muscle weakness or elevation of serum muscle enzymes [12, 13]. Furthermore, our patient had elevated anti-MDA5 antibodies which are strongly associated with CADM [14].

**Figure 4:**
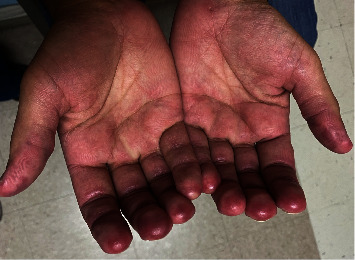
Erythematous fingertip papules.

He was initially treated with prednisone 40 mg orally daily and tacrolimus cream 0.1% twice daily on the affected areas. Two months later, RPC manifestations (arthritis, auricular chondritis, and nasal chondritis) resolved. However, gottron papules and subcutaneous nodules persisted, and he began presenting with painful fingertip papules (Figure 4). Cyclophosphamide 50 mg orally twice daily was added, but in the following 6 weeks, he had minimal improvement for which cyclophosphamide dose was increased to 50 mg orally three times daily. Six months later, due to persistence of skin lesions, cyclophosphamide was discontinued and switched to azathioprine 50 mg orally twice daily and hydroxychloroquine 200 mg orally twice daily. Three months later, he continued with recalcitrant skin manifestations; thus, he was treated with intravenous rituximab 1 gram every two weeks with two doses.

**Figure 5:**
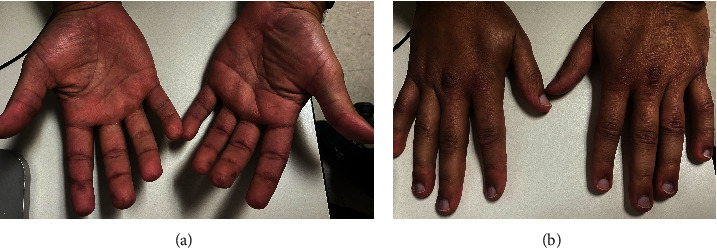
Resolution of (a) fingertip papules and (b) gottron papules after rituximab therapy.

Three months after rituximab treatment, gottron papules, subcutaneous nodules, and fingertip papules resolved (Figure 5). Azathioprine and hydroxychloroquine were continued, and prednisone dose was decreased to 20 mg without experiencing a reactivation of his disease. During the follow-up, he did not present further manifestations of RPC and CADM.

## 3. Discussion

We present a case of a young man with hypothyroidism who developed concurrent RPC and CADM. Initially, RPC resolved with corticosteroid therapy but continued with CADM skin manifestations requiring more aggressive immunosuppressive therapy with cyclophosphamide followed by azathioprine and hydroxychloroquine. He persisted with refractory skin manifestations for which he was treated with rituximab having a good clinical response. The association of RPC with idiopathic inflammatory myopathy is very unusual. Only few reports have described the association of RPC with DM.

About one third of RPC patients have associated diseases being systemic vasculitis, rheumatoid arthritis, Hashimoto thyroiditis, and myelodysplastic syndrome the most common [2, 5, 7]. Less frequently, RPC has been observed in patients with solid tumors [15]. Interestingly, positive ANA, which may occur in up to two thirds of patients with RPC, strongly suggests the presence of an autoimmune disease or a hematologic disorder/malignancy [6, 16, 17]. Our patient had positive ANA but extensive work-up for malignancy was negative.

CADM is a variant of DM characterized by the presence of classic DM cutaneous manifestations but without muscle weakness or elevated serum muscle enzymes. In addition to the presence of CADM features, our patient had elevated anti-MDA5 antibodies. CADM patients with high-titer anti-MDA5 antibodies have a high risk of developing rapidly progressive interstitial lung disease and severe cutaneous manifestations [18, 19]. To improve their survival, patients with CADM and positive anti-MDA5 are usually treated with aggressive immunosuppressive therapy. In addition to corticosteroid therapy, azathioprine, mycophenolate mofetil, tacrolimus, and cyclophosphamide have been used in these patients [20, 21]. Rituximab therapy has proven to be effective in resistant disease [22]. Our patient presented with several skin manifestations linked to anti-MDA5 antibodies, but he did not develop symptoms, signs, or imaging features suggestive of interstitial lung disease. It is plausible that early treatment with immunosuppressive drugs prevented this serious complication.

Including ours, only four cases have been reported of coexistent RPC and DM [7, 23]. However, our case is the first to report RPC presenting with CADM. Three out of four patients were men with ages between 30 and 50 years. Nguyen et al. described a 31-year-old man who developed muscle weakness, gottron papules, heliotrope rash, and periorbital edema. This patient was diagnosed with DM with concomitant RPC [24]. Two cases were reported by Frances et al, one male and one female with a mean age of 50. One patient also had myelodysplastic syndrome and the other had psoriasis [7]. DM cutaneous manifestations occurred at least 2 years before developing RPC. A plausible explanation for the lack of reports on the coexistence of these conditions is that both are extremely rare and that treatment of one condition could possibly prevent the occurrence of the other since the immunosuppressive drugs used to treat these diseases are similar.

The coexistence of RPC with DM is not unexpected because both entities share similar immunopathogenic mechanisms. First, CD4+ T cells appear to have an important role in the pathogenesis of both autoimmune diseases. In RPC, inflammatory infiltrates primarily composed of CD4+ T cells, macrophages, and plasma cells are found in the perichondrium of affected tissues [25]. In DM, the immune response is directed to the endothelium of capillaries and small blood vessels in which inflammatory infiltrates predominantly comprising CD4+ T cells and macrophages are found [26]. Second, in both conditions T-helper 1 (Th1)/T-helper 2 (Th2) imbalance occurs, enhancing the expression of INF-*γ*, IL-2, and IL-12 [27]. Third, a role of immune complexes has been suggested as immune and complement deposits are observed in affected tissues both in RPC and DM [25, 26]. Finally, recent studies have shown an absolute reduction of regulatory T cells in patients with RPC and inflammatory muscle disease compared to healthy controls [28–30].

In summary, we present a young adult who developed concurrent RPC and CADM. Although the coexistence of these diseases is very rare, it is important to maintain a high level of awareness of both diseases. Prompt detection and treatment of RPC could prevent destruction of cartilage structures, cardiac complications, and hematologic disorders such as myelodysplastic syndrome. On the other hand, delay of early recognition of CADM can cause serious complications including the development of rapidly progressive interstitial lung disease. Thus, early diagnosis of these diseases is critical to initiate effective therapy and avoid long-term complications.

## Data Availability

The data used to support the findings of this study are available from the corresponding author upon request.
